# Sugar-Free Milk Chocolate as a Carrier of Omega-3 Polyunsaturated Fatty Acids and Probiotics: A Potential Functional Food for the Diabetic Population

**DOI:** 10.3390/foods10081866

**Published:** 2021-08-12

**Authors:** Andrea R. Gómez-Fernández, Paulinna Faccinetto-Beltrán, Norma E. Orozco-Sánchez, Esther Pérez-Carrillo, Luis Martín Marín-Obispo, Carmen Hernández-Brenes, Arlette Santacruz, Daniel A. Jacobo-Velázquez

**Affiliations:** 1 Tecnologico de Monterrey, Escuela de Ingeniería y Ciencias, Av. General Ramón Corona 2514, Zapopan 45201, Mexico; andrea.rebeca.gf@gmail.com (A.R.G.-F.); paulinnafb1995@gmail.com (P.F.-B.); 2 Escuela Mexicana de Confitería y Chocolatería, Melchor Ocampo 926, San Luis Potosi 78280, Mexico; ccinvestigacionc@gmail.com; 3 Tecnologico de Monterrey, Escuela de Ingeniería y Ciencias, Av. Eugenio Garza Sada 2501, Monterrey 64849, Mexico; perez.carrillo@tec.mx (E.P.-C.); lmmarin@live.com.mx (L.M.M.-O.); chbrenes@tec.mx (C.H.-B.); asantacruz@tec.mx (A.S.)

**Keywords:** sugar-free, sweeteners, isomalt, stevia, milk chocolate, functional foods

## Abstract

Chocolate is an adequate matrix to deliver bioactive ingredients. However, it contains high sugar levels, one of the leading causes of chronic degenerative diseases. This work aimed to evaluate the effects of milk chocolate reformulation with alternative sugar sweeteners (Sw; isomalt + stevia), probiotics (Prob), and ω-3 polyunsaturated fatty acids (PUFAs) on its physicochemical properties and consumers’ acceptability. *Lactobacillus plantarum* 299v (L. p299v) and *Lactobacillus acidophilus* La3 (DSMZ 17742) were added as Prob strains, and fish oil (FO) was added as the source of ω-3 PUFAs. Prob addition resulted in chocolates with >2 × 10^7^ colony forming unit (CFU) per serving size (12 g). Except for Prob, a_w_ values of all treatments were <0.46. Sw and Sw + Prob presented the nearest values to the control in hardness, whereas Sw without FO increased fracturability. FO, Sw + FO, and Sw + Prob + FO contained 107.4 ± 12.84, 142.9 ± 17.9, and 133.78 ± 8.76 mg of ω-3 PUFAs per chocolate, respectively. Prob + FO increased the resistance of chocolate to shear stress, while Sw + FO showed a similar flow behavior to the control. The consumers’ acceptability of Sw + Prob chocolate was adequate, while Sw + Prob + FO had higher acceptability than Prob + FO. Health benefits of reformulated milk chocolates requires further assessment by in vitro, in vivo and clinical studies.

## 1. Introduction

Consumption of foods high in sugar is associated with the development of metabolic syndrome, which is defined as a collection of physiological, biochemical, and clinical factors, and is one of the leading causes of death worldwide [1]. Therefore, there is a need to develop new sugar-free products. Sweeteners are sugar substitutes, with natural sweeteners being more accepted in the market [2]. Additionally, there is an interest in the addition of bioactive ingredients to food formulations, in order to obtain food products that provide health benefits, including the prevention and treatment of diseases related to metabolic syndrome [3]. The term nutraceutical was coined in 1989 by Stephen DeFelice from the words “nutrition” and “pharmaceutical”, and he defined it as a food or part of a food that provides health benefits, including the prevention and treatment of disease beyond basic nutritional functions [4]. Recently, the term nutraceutical was revisited to separate the concept of food supplements and nutraceuticals [5]. Food supplements are food-derived products that compensate the lack of specific components (i.e., vitamins and minerals) in the daily diet and/or can exert a beneficial effect on health without any proven biological effect. On the other hand, nutraceuticals should have a proven beneficial pharmacological effect as a requirement [5]. In practical terms, as stated by Santini and Novellino, nutraceuticals should go beyond the diet, before the drug [5].

One of the food products experiencing more dynamic changes through this healthy demand is chocolate, since it represents 60% of the world’s confectionery market and is liked by adults and children due to its sweet taste and pleasant mouthfeel [3,6]. Sugar-free chocolates usually use a combination of sweeteners with high sweet power, such as stevia (Stev), and sweeteners as bulking agents, such as isomalt (Iso) [7]. Both sweeteners (Stev and Iso) are considered prebiotics [8,9]. Prebiotics are defined as non-digestible food ingredients that are metabolized by gut microbiota, improving host health [10]. Additionally, Stev is reported to exert beneficial effects on type 2 diabetes since this molecule interacts with intestinal and pancreatic cells, improving glucose uptake and helping to maintain glucose homeostasis [2,11].

The consumption of fish oil (FO) has been related to decreasing the risk of type 2 diabetes and other coronary diseases due to its high content of ω-3 PUFAs, such as eicosapentaenoic acid (EPA) and docosahexaenoic acid (DHA) [12,13]. The World Health Organization [14] recommend a consumption of 250–500 mg per day of combined EPA and DHA for healthy adults. Furthermore, several studies suggest that dietary ω-3 PUFAs from FO improve insulin sensitivity or reduce the incidence of type 2 diabetes thorough inhibition of adipose tissue inflammation [15].

Another bioactive ingredient that can be used to improve the health of the diabetic and non-diabetic populations are probiotics, since their consumption modulates gut microbiota [16]. Probiotics are defined as live microorganisms that confer health-promoting properties when administrated in adequate amounts to the host [17]. In this context, *Lactobacillus* *platarum* and *Lactobacillus acidophilus* have demonstrated to improve the health of type 2 diabetes patients by balancing the gut microbiota [18,19].

Chocolate could be an adequate vehicle for the delivery of probiotics and ω-3 PUFAs due to its main ingredients (cocoa butter, cocoa paste, soy lecithin, and milk) that generate a food matrix with low water activity, low oxygen tension, and low moisture permeability [20]. In addition, microencapsulation of probiotics provides double protection due to the covalently or ionically crosslinked polymer networks that enclose bacterial cells [21]. However, there are few reports in the literature on the development of functional sugar-free chocolates that could be consumed by the diabetic population.

The milk chocolate system comprises solid particles (cocoa, sugar, and milk powder) dispersed in the fat phase (cocoa butter). The composition of these ingredients affects the final sensory properties and rheological behavior as a fluid mass. To obtain high-quality products, the determination of these properties in chocolate manufacture must be well-defined to obtain the right palatable products and fulfill consumers’ preferences [22]. Rheological properties affect the final texture of chocolates, which plays a crucial role in the confectionery industry’s elaboration process [23]. For instance, if chocolate viscosity is too low, the texture would not be optimal, and if it is too high, bubbles may appear in the molded tablet. In addition to modifying texture, viscosity also affects the flavor of chocolate because the taste depends on the order and rate of contact, which is dependent on viscosity and melt rate. Chocolate rheology is usually determined by yield stress and apparent viscosity parameters. Yield stress provides information related to the transition behavior from elastic to viscous deformation. Furthermore, sensory evaluation is also a key element to evaluate the elaboration process of chocolate and ensure high-quality products that reach consumers’ preferences [24].

The objective of this study was to evaluate the effect of sugar substitution, probiotics and ω-3 PUFAs addition on the physicochemical properties and consumers’ acceptability of milk chocolate. Sugar was replaced by isomalt (Iso) and stevia (Stev), whereas the probiotics (Prob) strains added were *Lactobacillus plantarum* 299v (L. p299v) and *Lactobacillus acidophilus* La3 (DSMZ 17742). Furthermore, fish oil (FO) was used as a source of ω-3 PUFAs.

## 2. Materials and Methods

### 2.1. Bacterial Strains and Chemicals

Probiotic strains *Lactobacillus plantarum* 299v (L. p299v) and *Lactobacillus acidophilus* La3 (DSMZ 17742) were obtained from the German Collection of Microorganisms and Cell Cultures (DSMZ, Braunschweig, Germany) and the American Type Culture Collection (ATCC, Manassas, VA, USA), respectively. Sodium alginate was purchased from Deiman (Guadalajara, JAL, Mexico) and food-grade maltodextrin was obtained from Best Ingredients (Monterrey, NL, Mexico). Alkalinized cocoa paste, alkalinized cocoa, cocoa butter, whey powder, soy lecithin, polyglycerol polyricinoleate (PGPR), NaCl, vanilla, and sugar were obtained from Escuela Mexicana de Confitería y Chocolatería (San Luis Potosí, SLP, Mexico). Isomalt low moisture powder fine (LMPF) was obtained from Palsgaard Industry de México S de RL de CV (San Luis Potosí, SLP, Mexico). Stevia was obtained from Grupo Químico Amillán S.A. de C.V. (Zapopan, JAL, Mexico). Fish oil (Omega Pure^®^) was purchased from America Alimentos S.A. de C.V. (Zapopan, JAL, Mexico). For the fatty acid methyl esters profile determination, toluene-hexane mixture (1:1 *v*/*v*), undecanoic acid (100 ppm), and external standard fatty acid mixtures GLC 566 (39 fatty acid methyl esters) were purchased from Nu Chek Prep Inc (Elysian, MN, USA). Finally, for microbiological determinations, reconstituted skim milk (Svelty, Nestlé^®^) was obtained from a local market, whereas Violet Red Bile Agar (VRB agar), potato Dextrose Peptone Agar (DP agar), Xylose Lysine Deoxycholate Agar (XLD agar), Salmonella Shigella Agar (SS agar), Tetrathionate Broth Base, Rappaport Vassiliadis Broth, VRBA agar, and MRS agar were obtained from Sigma-Aldrich^®^ (St. Louis, MO, USA).

### 2.2. Bacterial Strains’ Propagation, Microencapsulation, and Viability Assessment

Bacteria were propagated by inoculating an aliquot (100 μL) from a stock of *Lactobacillus plantarum* 299v (L. p299v) and a stock of *Lactobacillus acidophilus* La3 (DSMZ 17742) in 10 mL of MRS broth, which was incubated at 37 °C in a Shel lab 1535 incubator (VWR, Randor, PA, USA) for 16 h under aerobic conditions. Then, propagation was scaled-up to a final volume of 800 mL under the same incubation conditions. Bacteria cells were harvested by centrifugation (at 10,000× *g*, 25 °C for 15 min). Cell pellets were washed in peptone water (0.1% peptone, 0.85% NaCl, pH 7) and resuspended in a final volume of 30 mL in peptone water.

Suspended cells were added to 750 mL of microencapsulation mix (10% *w*/*v* maltodextrin, and *w*/*v* 2% food-grade alginate) and spray-dried (ADL 311S, Yamato Scientific Co., Ltd., Santa Clara, CA, USA) at 130 °C inlet, 60 °C outlet, and 0.13 MPa. The viability of probiotics was determined by homogenizing the powder with microencapsulated probiotics (0.1 g) or the chocolates with added probiotics (1 g), with 90 mL of peptone water preheated at 37 °C in a stomacher (IUL Instruments, Barcelona, Spain) for 90 s. Proper dilutions (10^4^, 10^6^, and 10^8^) of each replicate were plated twice on MRS agar and incubated at 37 °C for 48 h, aerobically.

### 2.3. Chocolate Preparation

Milk chocolates were prepared in a confectionery pilot plant factory as previously described [25]. Chocolates were formulated to develop a sugar-free product rich in *Lactobacillus plantarum* 299v (L. p299v)*, Lactobacillus acidophilus* La3 (DSMZ 17742), and ω-3 PUFAs (EPA and DHA). Eight milk chocolate formulations were tested using the same base (alkalized cocoa paste 12%, natural cocoa powder 3%, cocoa butter 26%, whole milk powder 13%, skim milk powder 10%, soy lecithin 0.3%, PGPR 0.2%, NaCl 0.08%, and vanilla 0.003% *w/w*). Sugar was replaced with a mixture of Iso and Stev as sweeteners (Table 1). Likewise, FO and probiotics (*L. plantarum* 299v and *L. acidophilus* La3) were added as indicated in the formulations shown in Table 1.

Each chocolate formulation was produced by the following procedure: (1) melting and heating, (2) coaching, (3) refining, (4) tempering, and (5) molding. In the melting step, a water bath at 40 °C was used; for the coaching and refining steps, the temperature was 25 °C, and the duration was 24 h using a chocolate refiner (Premier, Diamond Custom Machines Corp., Hillsborough Township, NJ, USA). The tempering step followed three changes of temperature. The first stage of tempering was maintained at 45 °C to melt fat crystals (3–5 min); then, in the second stage, chocolate was cooled at 27 °C under manual agitation using a spatula (3–5 min), and finally, chocolate was reheated to 29 °C. Chocolate formulations were molded at 14 °C for 1 h and stored at 11 °C until analysis. FO and microencapsulated probiotics were added to chocolate after tempering at 29 °C at a ratio of 1 × 10^13^ UFC/g, resulting in chocolates with 2 × 10^7^ CFU per serving size (12 g).

### 2.4. Water Activity, Color, Texture, and Rheological Determinations

Water activity a_w_ of chocolate samples was measured using a water activity meter (Aqualab CX-2, Decagon Divices Inc., Pullman, WA, USA) at 25 °C using 3.0 g of the samples previously homogenized with a grinder (80350R, Hamilton Beach, Glen Allen, VA, USA). The color was determined with a spectrophotometer cm-600d (Konica Minolta Inc., Tokyo, Japan). Colorimetric parameters obtained (CIE L*, a*, and b*) were used to calculate the whiteness index (WI*) value, as indicated in Equation (1):

(1) WI*=100−[(100−L*)2+a*2+b*2]12

Treatments: Control = milk chocolate formulation, Prob = milk chocolate + probiotics, FO = milk chocolate + fish oil, Prob + FO = milk chocolate + probiotics + fish oil, Sw = sugar-free chocolate formulation (with added isomalt + stevia as sweeteners), Sw + Prob = sugar-free chocolate + probiotics, Sw + FO = sugar-free chocolate + fish oil, Sw + Prob + FO = sugar-free chocolate + probiotics + fish oil.

Hardness and fracturability (N) of the samples were determined using a texture analyzer (TVT 6700, Perten Instruments, Sydney, NSW, Australia) equipped with a cylinder probe (height 45 mm, diameter 3 mm). The conditions used were: sample height: 8 mm; starting distance from sample: 5 mm; compression: 2 mm; initial speed: 0.5 mm/s; test speed: 0.5 mm/s; retract speed: 10 mm/s; trigger force: 5 g; data rate: 500 pps, at 25 °C [25,26,27]. Five replicates of each treatment were evaluated.

Rheological experiments (flow behavior, stress sweep, and frequency sweep test) were carried out with a previously reported protocol [25,28]. A controlled stress rheometer (Physica MCR 101, Anton Paar, Ostfildern, Germany) fitted with a parallel plate geometry (PP25/S, 24.973 mm diameter, 1.0 mm gap) was used. Chocolate samples were melted in a water bath at 35 °C and poured on the bottom plate based on the methodology previously reported [25,28].

### 2.5. Fatty Acid Methyl Esters (FAMEs) Profile

Chocolate fat was extracted following the AOAC 948.22 Soxhlet method, using ethyl ether as the extraction solvent [29]. For each formulation, fat extraction was performed in triplicate from the chocolate bars (12 g). A sample of extracted fat (5 mg) was dissolved in a toluene-hexane mixture (0.6 mL, 1:1 *v*/*v*). Undecanoic acid (100 ppm) was added to samples as an internal standard for quantification. Subsequently, samples were derivatized using methanol-sulfuric acid (1 mL, 93:7 *v*/*v*) in capped vials placed in a water-bath (80 °C, 60 min). Thereafter, the samples were chilled, and the FAMEs were extracted with hexane and volume-adjusted (2 mL) for chromatographic analysis.

FAMEs profile was determined on a GC Agilent 6850A gas chromatograph coupled with a flame ionization detector (GC-FID, Agilent Technologies Inc., Santa Clara, CA, USA). The chromatography column employed was a fused-silica SP-2380 capillary column (100 m × 0.25 mm i.d., 0.2 µm film thickness, Supelco, Bellefonte, PA, USA). The chromatographic setup and FAMEs’ identification and quantification were performed as previously reported by Faccinetto-Beltrán et al. [25]. Quantification for each compound and the total amount of fatty acids (FAs) were calculated by the AOAC method 996.06. Concentration of FAs were expressed as mg of each individual FA per 100 g of product on a fresh weight (FW) basis.

### 2.6. Sensory Evaluation

A sensory acceptability test was performed using the 9-point hedonic scale to assess the consumers’ acceptability of chocolate formulation. A total of 223 students and staff (59% male and 41% female) from Tecnológico de Monterrey (Monterrey, NL, México) that consumed chocolate at least once a week were selected for the study, with ages ranging between 17 and 21 years old. Each chocolate sample was provided with a different random three-digit number. The samples were provided in different orders. Participants were asked to eat the chocolate samples one at a time, drink water, and eat a cookie with a plain flavor before the evaluation and between testing different samples. For each chocolate, the participants were requested to evaluate the attributes of appearance, flavor, texture, and overall acceptability using a 9-point hedonic scale ranging from 1 to 9: 1 = “dislike extremely,” 2 = “dislike very much,” 3 = “dislike moderately,” 4 = “dislike slightly,” 5 = “neither like nor dislike,” 6 = “like slightly,” 7 = “like moderately,” 8 = “like very much,” and 9 = “like extremely.”

To determine the microbial safety of chocolate samples before sensory evaluation, chocolate formulations were analyzed for total coliforms, yeast, molds, and *Salmonella* spp., according to methods previously reported in the literature [30,31,32]. Briefly, 10 g of each chocolate sample was introduced into a sample bag (Whirl-Pak^®^, Nasco, Fort Atkinson, WI, USA), diluted with sterile peptone water (0.1 % peptone, 0.85% NaCl, pH 7), and homogenized for 2 min in a stomacher. Triplicate counts were performed for all dilutions. Total coliforms were determined using violet red bile agar and incubated at 37 °C for 24 h. Fungi and molds were grown in potato dextrose peptone agar and incubated at 25 °C for 5 days. All chocolates presented <10 CFU/mL for total coliforms, fungi, and molds.

For *Salmonella* spp. analysis, 25 g of chocolate sample was placed in 225 mL of reconstituted skim sterilized milk for 60 min at 25 °C. Then, 1 mL of each sample was put in 10 mL of Vassiliadis-Rappaport and in 10 mL of tetrathionate for 24 h. *Salmonella* spp. counts were performed in XLD agar and SS agar. Chocolate formulations were free of *Salmonella* spp., and thus, all chocolates were safe for human consumption and suitable for sensory evaluations (Ethics ID: CSERMBIGDL-002).

### 2.7. Statistical Analysis

Results are expressed as mean ± standard error of three independent measurements unless otherwise indicated. Data were analyzed with full factorial analysis of variance to evaluate main effects and interactions, followed by the LSD test to determine significant differences among groups (*p* < 0.05), using JMP software version 14.3.0 (SAS Inst. Inc., Cary, NC, USA).

## 3. Results and Discussion

### 3.1. Probiotics Viability

Microencapsulation by spray-drying is a common technology to protect the viability of probiotics [33]. In the present study, maltodextrin (10%, *w*/*v*) and sodium alginate (2%, *w*/*v*) were used as bacteria-protecting ingredients to generate powders with microencapsulated probiotics. Spray-drying microencapsulation resulted in powders with 7 × 10^13^ CFU/g and 1 × 10^14^ CFU/g for *Lactobacillus plantarum* L299v and *Lactobacillus acidophilus* La 3, respectively. These results agree with previous reports that evaluated microencapsulation of probiotics with sodium alginate, demonstrating that it can be used as a heat protector agent for different probiotic strains, such as *L. rhamnosus, B. longum, L. salivarius, L. plantarum, L. acidophilus, L. paracasei, B. lactis* B1-O4, *B. lactis* Bi-07 [34], and *L. casei* [35]. Furthermore, the use of prebiotic agents such as maltodextrin, in addition to alginate, is recommended to generate a physical barrier with a symbiotic relationship [36]. In this tenor, previous reports have demonstrated that maltodextrin can be used as an effective microencapsulating protective agent for probiotics, reducing the caking and stickiness to the spray-dryer’s wall, increasing the free-flowing nature of the spray-dried powder [37], and exerting heat protection [38].

For all chocolate formulations, the addition of microencapsulated probiotics resulted in a product with ≥2 × 10^7^ CFU per portion (12 g). This value is in the range of the minimum count of probiotic bacteria intake (≥1 × 10^6^ CFU) recommended to have a beneficial effect [39,40]. Prior reports have shown that chocolate ingredients are suitable as a vehicle for probiotics [20,25,27]. For instance, the high total solids in milk chocolate, including fat and protein, generate a protective matrix for probiotics [40]. Furthermore, the low water activity (a_w_) and fat concentration in chocolate aid in preserving the viability of probiotic bacteria in an inactive state.

### 3.2. Physicochemical Properties of Sugar-Free Milk Chocolate Formulations with Added Probiotics and Fish Oil

#### 3.2.1. Water Activity (a_w_)

Water activity has an important role in the safety, quality, processing, shelf-life, texture, and sensory characteristics of confectionary products [3]. The a_w_ values for milk chocolate formulations are shown in Table 2. The control showed a_w_ = 0.46, which is in the threshold for a_w_ values of pathogenic microbial growth in foods. Sweetener addition (Sw), FO addition, and their combination (Sw*FO) showed a significant reduction in a_w_, whereas Prob addition impeded this effect. Water activity reduction by isomalt addition has been previously reported for sugar-free milk chocolate formulations, which has been attributed to its hygroscopic property [41]. FO addition generated chocolate formulations with lower a_w_ values. This phenomenon could be attributed to the degree of unsaturation in fatty acid, generating electric charges that affect the molecular interaction with water molecules [42].

#### 3.2.2. Whiteness Index (WI)

The WI indicates fat bloom formation [43]. Fat blooming plays a crucial role in the final structure, mechanical properties, appearance, quality, and marketability of chocolate products [44]. The effects of Sw, FO, and Prob addition as well as their interactions in the WI values of chocolate are shown in Table 2. Sw and Prob added alone (without sugar replacement) showed a significant increase on the WI value, whereas FO added alone did not affect the WI value. However, when FO was added to sugar-free chocolate (Sw + FO and Sw + Prob + FO), the individual effect of Sw and Prob on the increase in WI value was impeded.

The lower WI values observed in Sw treatments indicate that sugar replacement by sweeteners generates darker chocolates less prone to fat blooming. This result is in agreement with previous reports, where sucrose replacement with polyols, such as maltitol, xylitol, isomalt, and stevia, generated darker chocolates compared to their reference chocolate [45,46]. Particle size and distribution play an important role in instrumental color measurements. The tempering process of Sw and Sw + Prob chocolates could be responsible for the development of appropriate cocoa butter nucleation, generating more stable microparticle interaction due to the generation of adequate amounts and sizes of β V polymorphic form crystals [47].

On the other hand, Prob addition increased fat blooming predisposition in the formulation. These agreed with a report where the incorporation of *L. paracasei* to white chocolate formulation generated brighter chocolates as compared to the control [48]. The color of microencapsulated probiotic powder can explain this increase since probiotics could affect the particle size distribution in the chocolate matrix [49]. Since microcapsules are composed of carbohydrates such as maltodextrin, sugar bloom and fat bloom could be occurring. Sugar bloom is caused by absorption of moisture solubilizing sugar and then re-crystallized at the surface as a thin film of sugar crystals [50]. The fat bloom is distinguished from loss of gloss caused by larger crystals’ growth, causing scattering of the light, and the surface appears dull, due to an incorrect tempering [51]. Similar results from chocolate with added probiotics were obtained by Silva et al. [52]. The authors attributed this phenomenon to the addition of probiotics during the tempering process, which influences the recrystallization of lipids. However, the interaction of Sw and Prob shows a decrease in WI values, which could be related to the microstructure interaction between sweeteners, Prob, and other ingredients in the chocolate formulation.

#### 3.2.3. Texture

Hardness and fracturability are two texture parameters that have a direct correlation with the acceptability by consumers. Hardness represents the physical rigidity, whereas fracturability is associated with the maximum force for penetration [6,7]. Prob, FO, and sugar replacement (Sw) either evaluated alone or combined significantly reduced the hardness value of chocolate. The hardness decrease by FO addition could be attributed to the increase of PUFAs in the chocolate matrix, yielding a softer product that melts easier [53]. Lipids in chocolate represent the continuous phase in the chocolate emulsion, which governs the physical and the textural properties. The hardness of chocolate is affected by the extent and nature of the crystalline lipid phase, linked to the control of the proper polymorphic form controlled by tempering [47].

Probiotics’ addition also decreased the hardness value of chocolate. This result is in agreement with a previous report where the hardness of chocolate was evaluated in dark chocolates with and without probiotics [54]. The authors attributed the lower hardness values to the effect that microencapsulated probiotics’ addition could have on crystal formation during the tempering process. Furthermore, sugar replacement (Sw) also generated chocolates with lower hardness values. Polyols sweeteners such as isomalt and high-intensity sweeteners such as stevia affect the texture of chocolate due to their hydroxyl sites, which interact with intermolecular bonds between particles in chocolate [45,46].

Fracturability of chocolate was increased only when Prob and FO were added either alone or combined in sugar-free chocolates. For instance, treatments without sugar and without FO showed higher fracturability values as compared with the control. Interestingly, FO addition in sugar-free chocolates decreased fracturability, showing the lowest values among treatments (Table 2). Previous studies on physical analyses of chocolate formulations with added EPA/DHA in the triglyceride form resulted in a softer product as compared with the control, attributed to the high content of PUFAs, which contributes to the generation of a softer product with lower fracturability when sugar is replaced [55]. Other authors have suggested that process and product optimization could improve the texture of chocolates when the formulation has added FO or EPA/DHA as microencapsulated oil and powder, overcoming undesirable textural and physiological effects of FO addition [3,55].

Texture values presented herein are influenced by the tempering process, since properly tempered chocolate contains numerous β V polymorph crystals of cocoa butter that form a tight crystalline matrix, giving a high degree of hardness and fracturability. Besides, in milk chocolates, it is important to consider the effect of milk fat on cocoa butter crystallization since it can influence the modification of β V crystals to β’polymorph, which foment disorder in the emulsion matrix [6,47]. Therefore, the fatty acid composition affects liquid fat solidification, and thus the texture properties. The addition of isomalt in chocolate has been reported to increase hardness and fracturability [56]. However, the interaction between Sw and FO treatments decreased fracturability values, likely due to the increased concentration of PUFAs [3,55].

### 3.3. Rheological Analysis: Shear Stress, Apparent Viscosity, and Frequency Sweep Test

#### 3.3.1. Flow Behavior

Rheological characteristics of chocolate are directly related to the quality attributes of the product [23]. Viscosity plays an important role in texture, flavor, and mouthfeel. Likewise, flow properties can be perceived by consumers in flavor and mouthfeel, since the perceived taste depends on the melting rate [6].

**Figure 1:**
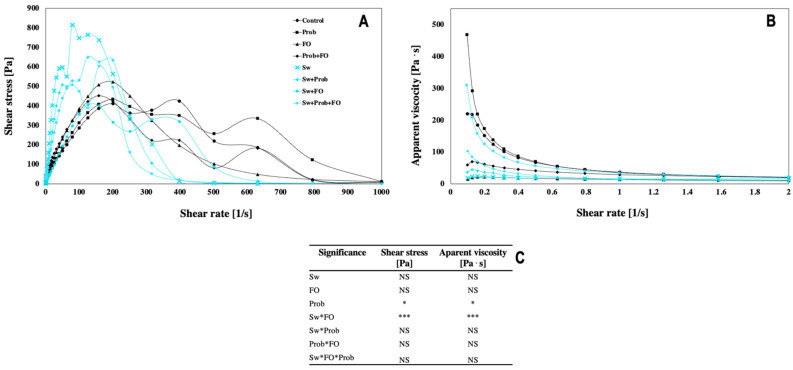
(**A**) Shear stresses were at a range of steady-shear rate 0.1 to 100 s^−1^ and temperature 35 °C. (**B**) Apparent viscosity was at a range of steady-shear rate 0.1 to 100 s^−1^ and temperature 35 °C. (**C**) Full factorial analysis of variance showing the main effects and interactions of the variables evaluated. Values represent the mean of 3 replicates. Asterisks indicate significant difference from a full factorial analysis of variance showing the main effects and interactions of the variables evaluated: * *p* < 0.05, *** *p* < 0.001. Sw, sweetener; FO, fish oil; Prob, probiotic; NS, non-significant. Treatments: Control = milk chocolate formulation, Prob = milk chocolate + probiotics, FO = milk chocolate + fish oil, Prob + FO = milk chocolate + probiotics + fish oil, Sw = isomalt + stevia, Sw + Prob = isomalt + stevia + probiotics, Sw + FO = isomalt + stevia + fish oil, Sw + Prob + FO = isomalt + stevia + probiotics + fish oil.

The variations of shear stress versus shear rate as well as apparent viscosity versus shear stress of milk chocolate formulations are shown in Figure 1. Probiotics’ addition induced a significant increase in shear stress and apparent viscosity values, whereas sugar replacement (Sw) and FO addition evaluated individually did not affect shear stress values or apparent viscosity values. However, the Sw*FO interaction significantly modified the rheological behavior of chocolate. Chocolates with added Prob showed a plunge more stable than the control (Figure 1A). Likewise, FO combined with Sw significantly modified the shear stress. For instance, Sw + FO treatment showed a similar flow behavior as compared with the control. Additionally, Prob showed the highest apparent viscosity values compared with the control and the other sucrose milk chocolate formulations (Figure 1B). Nevertheless, FO addition affected the apparent viscosity as well as the use of Sw. As the apparent viscosity decreased, the shear rate increased, which agrees with the pseudoplastic or shear-thinning nature of chocolate [22].

The higher apparent viscosity observed in the Prob treatment could be attributed to an increase in the size and number of solid particles in the chocolate formulation. A study conducted by Afoakwa et al. [49] showed that the increase of an average particle size resulted in a decrease of Casson plastic viscosity, shear stress, yield stress, and apparent viscosity. Furthermore, previous reports have demonstrated that the addition of lyophilized probiotics increased rheological parameters and negatively affected chocolate flow properties [57,58].

As described earlier, the content and type of ingredients, such as the incorporation of PUFAs, have a critical role in chocolate viscosity. For instance, FO addition in chocolates induced a decrease in the shear stress since the fat in chocolate recovers solid particles, allowing an easy flow [23]. Similar observations were reported by Konar et al. [3], who evaluated the addition of different sources of DHA/EPA, and the authors reported a decrease in shear stress.

On the other hand, sweeteners induced an increase in shear stress, indicating that sugar-free chocolate formulations did not reach a steady condition in their rheology (Figure 1A). Previous authors studied the rheology of chocolates with different added bulk sweeteners, including isomalt, and observed that the shear-thinning index changes between the control (chocolate with sucrose) and the different bulk sweeteners. Likewise, the authors concluded that each sweetener’s structure interacts with other particles in the chocolate matrix in each void space [28,47]. Void spaces between cocoa particles and cocoa butter allow optimal rheology. When the void space is too tight, the shear-thinning index is increased, and the viscosity is reduced. Similar behavior occurs when adding isomalt. However, the special molecular conformation of isomalt allows more void spaces, reducing the shear-thinning index [28].

#### 3.3.2. Mechanical Properties

**Figure 2:**
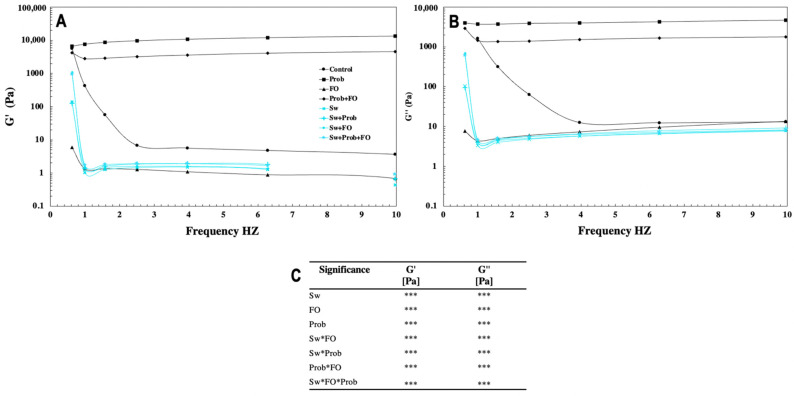
Frequency sweep test of chocolate at 35 °C with a linear viscoelastic region of 6 Pa. (**A**) Changes in storage modulus G′. (**B**) Changes in loss modulus G″. (**C**) Full factorial analysis of variance showing the main effects and interactions of the variables evaluated. Values represent the mean of 3 replicates. Asterisks indicate significant difference from a full factorial analysis of variance showing the main effects and interactions of the variables evaluated: *** *p* < 0.001. Treatments: Control = milk chocolate formulation, Prob = milk chocolate + probiotics, FO = milk chocolate + fish oil, Prob + FO = milk chocolate + probiotics + fish oil, Sw = isomalt + stevia, Sw + Prob = isomalt + stevia + probiotics, Sw + FO = isomalt + stevia + fish oil, Sw + Prob + FO = isomalt + stevia + probiotics + fish oil.

The mechanical spectra of chocolate samples are shown in Figure 2. G′ is an index of a sample’s elastic behavior and represents the deformation energy stored in the sample during the shear process. On the other hand, the G″ value measures the viscous component of a sample and compares the energy lost during the shear process [59]. The addition of Prob increased the storage modulus G′ over the loss modulus G”. On the other hand, FO addition and Iso + Stev showed a contrast effect on G′ and G” at the frequency range of 0.1 to approximately 70 Hz, indicating a liquid-like behavior of a weakly structured system.

Prob addition generated a structured system to the chocolate emulsion due to the results of G′ over G” presented herein. This behavior has been previously reported for milk chocolate [22,59]. Additionally, the combination of Prob with FO (Prob + FO) showed a similar result to Prob, meaning that Prob as an ingredient increased the stability of chocolate’s mechanical properties. Although the addition of Prob generated a strong matrix, when Prob was mixed with Sw (Sw + Prob) or Sw + FO (Sw + Prob + FO), a solid-like and more elastic formulation was observed mainly due to a higher solid fraction. On the other hand, the addition of FO generated a liquid-like behavior when it was combined with Sw. These results indicate that FO addition dominates the mechanical properties in sugar-free chocolates, such as Sw + FO and Sw + Prob + FO, because FO increased the number of fatty acids in the chocolate’s emulsion [53]. The fat content of chocolate determines the mass fraction of particulates, which governs the proximity of those particles to each other. Thus, if fat content increases, the distance between particles increases, resulting in a lower viscosity [47]. These observations are in agreement with the results obtained for chocolates with added FO. Furthermore, results of the substitution of sucrose by Sw in chocolates showed an unstable chocolate matrix, generating a deep increase of G′ and G″, as observed in Figure 2. This behavior is attributed to the higher solid volume fraction and lower density of isomalt, resulting in more flexible chocolates [28].

### 3.4. Fatty Acid Methyl Esters (FAMEs) Profile

The most widely available dietary source of EPA and DHA is cold-water oily fish or fish oil offered to consumers as a dietary supplement [55]. The chocolates formulated herein had 790 mg of FO added per serving size (12 g) of chocolate, expecting to obtain 200 mg of ω-3 PUFAs.

The fatty acid composition of chocolates with and without added FO are shown in Table 3. Likewise, the fatty acid composition of FO used as an ingredient for chocolate formulations is shown in the Supplementary Material (Table S1). FO contains a high amount of PUFAs (38,746.6 ± 45.8 mg per 100 g fish oil), of which ω-3 were the most abundant (34,712.6 mg ± 0.06 g per 100 g fish oil), with DHA (C_22:6_, 14,122.2 ± 27.0 mg/100 g) and EPA (C_20:5_, 12,862.1 ± 17.8 mg/100 g) being the major ω-3 PUFAs.

Chocolates without FO showed a low concentration of ω-3 PUFAs, mainly due to the presence of alfa linolenic acid in cocoa butter [53]. Additional fatty acids detected in the control and Prob treatment included linoleic acid, palmitic acid, stearic acid, and oleic acid, which are the primary fatty acids of cocoa butter [6,53,60]. FO addition resulted in a chocolate formulation with 107.4 ± 12.84 mg of ω-3 PUFAs per serving size (12 g). Interestingly, higher ω-3 PUFAs content was quantified when FO was added in sugar-free chocolate formulations (Sw + FO and Sw + Prob + FO) as compared with FO added alone, showing ω-3 PUFAs levels of 141.9 ± 17.9 mg and 133.8 ± 8.76 mg per 12 g of Sw + FO and Sw + Prob + FO formulations, respectively.

FO was added to the chocolate formulation to obtain 200 mg of ω-3 PUFAs per portion (12 g). However, results indicate that lower amounts were detected, indicating that ω-3 PUFAs were degraded during the chocolate-making process. Fatty acid degradation during the chocolate-making process can be attributed to lipid oxidation induced by low water activity and thermal treatment [3], which degrades EPA and DHA by breaking down the double bonds by oxidation [61,62].

### 3.5. Consumers’ Acceptability

A consumer acceptability test was performed to evaluate the appearance, taste, texture, and overall acceptability of chocolates, using a 9-point hedonic scale (Table 4).

FO addition significantly reduced the acceptability of chocolate for all the parameters evaluated. On the other hand, Prob did not affect the acceptability by consumers. These results are in agreement with previous reports, where probiotics’ addition did not affect the acceptability of chocolate [25,27,49]. FO addition mainly affected the acceptability of flavor and texture in FO and Prob + FO chocolates. This may be related to the fish odor present in fish oil. Interestingly, when FO was added to sugar-free formulations (Sw + FO and Sw + Prob + FO), chocolates showed higher acceptability as compared with formulations containing sugar and FO (FO and Prob + FO). This behavior can be explained by the fact that sugar can enhance flavors [63], and Sw chocolates have antioxidant properties (due to isomalt) that could protect FO from lipid oxidation [64].

Sugar-free chocolates showed lower flavor, texture, and overall acceptability values as compared with the control. The lower acceptability scores could be attributed to stevia’s bitter taste and to the changes in rheological and mechanical properties induced by Sw addition [65,66]. It is important to point out that sugar-free chocolates, and sugar-free chocolates with added probiotics, showed values in the acceptable range, indicating that they could be excellent candidates for commercialization.

## 4. Conclusions

In the present study, it was demonstrated that it is possible to formulate sugar-free milk chocolate formulations with added ω-3 PUFAs and probiotics, showing adequate acceptability by consumers. One of the drawbacks of the formulations evaluated was the decrease in acceptability by consumers when FO was added as an ingredient. Therefore, further studies should consider using lower concentrations of FO, or adding the ω-3 PUFAs from other sources, such as microalgae. The results presented herein support the idea that chocolate could be used as a good delivery system of bioactive ingredients, and thus further studies should evaluate the effect on these new chocolate formulations on the prevention of diseases through the evaluation of their efficacy by in vitro, in vivo and clinical studies.

## Figures and Tables

**Table 1:** Milk chocolate formulations added with probiotics and fish oil.

Ingredients	% Percentage in Each Formulation (*w*/*w*)							
	Control	Prob	FO	Prob + FO	Sw	Sw + Prob	Sw + FO	Sw + Prob + FO
Alkalinized cocoa paste	12.46	12.43	11.64	11.61	13.00	12.97	12.12	12.12
Natural cocoa	3.00	3.00	2.80	2.80	3.00	3.00	2.80	2.80
Cocoa butter	26.15	26.10	24.43	24.39	23.24	23.19	21.67	21.67
Whole milk powder	13.42	13.39	12.54	12.51	14.00	13.97	13.05	13.05
Skim milk powder	10.54	10.51	9.85	9.83	11.00	10.98	10.25	10.25
Soy lecithin	0.384	0.38	0.36	0.36	0.30	0.30	0.30	0.30
PGPR	0.192	0.19	0.18	0.18	0.20	0.20	0.19	0.19
NaCl	0.08	0.08	0.07	0.07	0.08	0.08	0.08	0.08
Vanilla	0.03	0.03	0.03	0.03	0.03	0.03	0.03	0.03
Sugar	33.75	33.68	31.53	31.47	-	-	-	-
Isomalt LMPF	-	-	-	-	35.12	35.05	32.95	32.74
Stevia	-	-	-	-	0.03	0.03	0.03	0.03
Probiotic	-	0.21	-	0.21	-	0.21	-	0.21
Fish oil	-	-	6.57	6.55	-	-	6.54	6.54

Abbreviations: Prob, probiotics; FO, fish oil; Sw, sweeteners; PGPR, polyglycerol polyricin; LMPF: low moisture powder fine.

**Table 2:** Water activity (a_w_), whiteness index (WI), and texture parameters’ (hardness and fracturability) values of sugar-free milk chocolate formulations with added probiotics and fish oil.

Sample	a_w_^a^	WI	Hardness ^b^ (N)	Fracturability ^b^ (N)
Control	0.46 ± 0.02 a	19.08 ± 0.99 de	3072.8 ± 93.6 a	2824.2 ± 117.5 b
Prob	0.47 ± 0.01 a	27.21 ± 0.29 a	2170.6 ± 198.3 c	2676.6 ± 129.8 b
FO	0.45 ± 0.01 a	20.73 ± 0.49 de	1644.6 ± 103.9 d	2834.2 ± 202.9 b
Prob + FO	0.45 ± 0.01 a	26.01 ± 0.26 ab	1719.8 ± 176.3 d	2823.8 ± 294.1 b
Sw	0.41 ± 0.01 b	24.68 ± 1.29 bc	2709.2 ± 140.5 b	3606.2 ± 96.8 a
Sw + Prob	0.40 ± 0.01 b	14.69 ± 1.41 f	2599.0 ± 103.6 b	3300.6 ± 101.9 a
Sw + FO	0.45 ± 0.01 a	18.95 ± 0.49 e	1241.2 ± 47.7 e	2031.6 ± 121.6 c
Sw + Prob + FO	0.46 ± 0.01 a	21.45 ± 1.47 cd	1545.2 ± 44.5 de	2454.2 ± 63.9 bc
Significance ^c^				
Sw	**	***	NS	NS
FO	*	NS	***	***
Prob	NS	*	NS	NS
Sw*FO	***	NS	NS	***
Sw*Prob	NS	***	*	NS
Prob*FO	NS	**	***	NS
Sw*FO*Prob	NS	***	NS	NS

W.I., white index; a_w_, water activity. Treatments: Control = milk chocolate formulation, Prob = milk chocolate + probiotics, FO = milk chocolate + fish oil, Prob + FO = milk chocolate + probiotics + fish oil, Sw = isomalt + stevia, Sw + Prob = isomalt + stevia + probiotics, Sw + FO = isomalt + stevia + fish oil, Sw + Prob + FO = isomalt + stevia + probiotics + fish oil. Values with different letters within the same column indicate a statistically significant difference by the LSD test (*p* < 0.05). ^a^ Values represent the mean of 3 replicates with their standard error. ^b^ Values represent the mean of 5 replicates with their standard error. ^c^ Asterisks indicate significant difference from a full factorial analysis of variance showing the main effects and interactions of the variables evaluated: * *p* < 0.05, ** *p* < 0.01, *** *p* < 0.001. Sw, sweetener; FO, fish oil; Prob, probiotic; NS, non-significant.

**Table 3:** Fatty acid profile content (mg fatty acid per 100 g sample FW^−1^) of fish oil source, milk chocolate with added probiotics and fish oil samples, and sugar-free milk chocolates with added probiotics and fish oil samples.

Fatty Acid	Chocolate Samples							
	Control	Prob	FO	Prob + FO	Sw	Sw + Prob	Sw + FO	Sw + Prob + FO
Octanoic acid (C8:0)	46.55 ± 4.61 b	55.47 ± 2.67 ab	58.027 ± 7.67 ab	56.19 ± 4.20 ab	53.46 ± 4.78 ab	62.021 ± 5.08 ab	65.36 ± 8.81 a	613.28 ± 6.05 ab
Decanoic acid (C10:0)	10.48 ± 1.70 b	12.84 ± 0.93 ab	16.55 ± 3.33 ab	14.20 ± 1.78 ab	13.89 ± 1.61 ab	16.02 ± 2.69 ab	10.4792 ± 1.71 a	104.79 ± 1.70 a
Lauric acid (C12:0)	8.02 ± 0.94 c	9.91 ± 0.75 bc	14.33 ± 1.23 abc	16.33 ± 3.34 ab	12.41 ± 1.12 abc	13.89 ± 3.61 abc	17.80 ± 2.41 a	165.47 ± 2.92 a
Myristic acid (C14:0)	33.48 ± 0.76 c	39.85 ± 0.31 c	261.24 ± 14.08 b	265.52 ± 30.74 b	46.89 ± 1.38 c	78.45 ± 32.45 c	354.92 ± 26.85 a	3208.28 ± 18.87 ab
Pentadecanoic acid (C15:0)	9.22 ± 0.44 c	10.53 ± 0.25 c	28.57 ± 1.48 b	29.23 ± 3.04 b	11.79 ± 0.84 c	13.51 ± 2.85 c	38.37 ± 3.15 a	365.43 ± 2.9 a
Palmitic acid (C16:0)	5412.38 ± 61.76 bc	6390.28 ± 63.55 a	5061.02 ± 242.55 c	4739.24 ± 326.71 c	6117.75 ± 198.24 ab	6237.88 ± 389.55 ab	6098.85 ± 332.69 ab	64,200.62 ± 440.60 a
Heptadecanoic acid (C17:0)	43.37 ± 0.45 c	49.97 ± 0.16 bc	54.58 ± 3.12 b	51.79 ± 3.55 bc	48.62 ± 1.71 bc	49.84 ± 5.11 bc	67.13 ± 4.22 a	674.77 ± 5.82 a
Stearic acid (C18:0)	6684.21 ± 2.10 b	7846.70 ± 79.76 a	5617.02 ± 225.79 c	5190.71 ± 335.91 c	7476.05 ± 246.78 ab	7566.89 ± 421.72 ab	6785.18 ± 354.72 b	72,410.12 ± 507.05 ab
Arachidic acid (C20:0)	220.47 ± 2.10 b	257.99 ± 2.45 a	181.37 ± 7.51 c	164.50 ± 10.85 c	249.25 ± 8.49 ab	251.35 ± 13.82 ab	227.57 ± 11.71 ab	2423.21 ± 16.91 ab
Behenic acid (C22:0)	40.01 ± 0.16 bc	46.55 ± 0.27 a	35.98 ± 1.43 cd	31.59 ± 2.16 d	45.18 ± 1.22 ab	46.05 ± 2.48 ab	45.35 ± 2.64 ab	471.32 ± 4.05 a
Lignoceric acid (C24:0)	25.69 ± 0.30 bcd	29.93 ± 3.52 abc	23.82 ± 1.83 cd	22.92 ± 1.19 c	31.81 ± 2.09 ab	30.36 ± 1.49 abc	33.14 ± 2.44 a	317.14 ± 3.39 ab
Myristoleic acid (C14:1)	N.D.	N.D.	N.D.	N.D.	N.D.	N.D.	N.D.	N.D.
Palmitoleic acid (C16:1)	50.39 ± 1.30 c	59.17 ± 0.00 c	336.49 ± 18.50 b	330.66 ± 36.51 b	57.69 ± 1.96 c	77.91 ± 23.23 c	453.92 ± 30.49 a	419.48 ± 24.94 a
Oleic Acid (C18:1)	6329.61 ± 52.79 c	7403.02 ± 0.07 a	4967.76 ± 194.57 d	4563.29 ± 265.40 d	7118.32 ± 246.68 abc	7211.03 ± 373.29 ab	6473.06 ± 341.50 bc	6854.78 ± 433.29 abc
Vaccenic acid (C18:1)	65.78 ± 1.12 c	76.13 ± 0.00 c	129.99 ± 65.57 b	124.2 ± 12.02 b	72.46 ± 2.90 c	77.29 ± 9.70 c	168.56 ± 10.77 a	165.24 ± 11.53 a
Eicosenoic acid (C20:1)	190.24 ± 0.31 c	13.11 ± 0.00 c	18.511 ± 1.10 b	17.89 ± 1.79 b	12.57 ± 0.44 c	14.46 ± 2.20 bc	26.20 ± 2.11 a	25.61 ± 2.05 a
Nervonic acid (C24:1)	N.D.	N.D.	18.511 ± 1.10 a	15.93 ± 1.48 a	N.D.	N.D.	18.51 ± 3.11 a	11.09 ± 1.88 b
Linoleic acid (C18:2)	654.58 ± 5.59 b	767.95 ± 8.11 a	407.71 ± 19.88 c	391.90 ± 19.86 c	733.25 ± 21.76 ab	730.29 ± 28.96 ab	687.41 ± 40.22 ab	736.66 ± 49.90 ab
Gamma Linolenic acid (C18:3)	N.D.	N.D.	9.55 ± 1.49 a	8.65 ± 0.75 a	N.D.	N.D.	10.83 ± 1.45 a	11.46 ± 2.28 a
Alpha Linolenic acid (C18:3)	48.60 ± 0.66 c	58.40 ± 0.94 c	79.41 ± 4.60 b	73.60 ± 6.67 b	53.22 ± 1.51 c	55.44 ± 3.47 c	114.22 ± 7.76 a	112.20 ± 6.96 a
Stearidionic acid (C18:4)	N.D.	N.D.	90.37 ± 6.48 b	87.67 ± 11.05 b	N.D.	N.D.	114.66 ± 6.62 a	109.23 ± 7.67 a
Eicosadienoic acid (C20:2)	N.D.	N.D.	28.21 ± 2.38 a	26.51 ± 2.89 a	N.D.	N.D.	17.69 ± 2.24 b	18.40 ± 2.98 b
Homo-gamma-linolenic acid (C20:3)	N.D.	N.D.	9.012 ± 0.82 b	8.86 ± 0.55 b	N.D.	N.D.	10.63 ± 1.44 ab	12.04 ± 1.39 a
Dihomogamma linolenic acid (C20:3)	N.D.	N.D.	13.99 ± 1.85 a	11.07 ± 1.20 a	N.D.	N.D.	12.57 ± 1.47 a	13.87 ± 2.02 a
Arachidonic acid (C20:4)	N.D.	N.D.	22.92 ± 2.66 b	22.75 ± 2.02 b	N.D.	N.D.	31.18 ± 2.73 a	28.57 ± 2.26 a
Eicosapentaenoic acid (C20:5)	N.D.	N.D.	316.75 ± 20.57 b	305.6 ± 35.34 b	N.D.	N.D.	421.21 ± 32.36 a	309.91 ± 22.28 a
Docosapentaenoic acid n-6 (C22:5)	N.D.	N.D.	8.37 ± 20.57 b	9.02 ± 0.66 b	N.D.	N.D.	14.98 ± 2.19 a	14.84 ± 0.87 a
Docosapentaenoic acid n-3 (C22:5)	N.D.	N.D.	42.39 ± 6.66 b	46.33 ± 5.10 b	N.D.	N.D.	64.63 ± 5.93 a	52.013 ± 7.22 ab
Docosahexaenoic acid (C22:6)	N.D.	N.D.	351.83 ± 22.20 b	341.07 ± 43.00 b	N.D.	N.D.	463.481 ± 31.99 a	436.57 ± 27.53 a
Total ω-3	48.61 ± 0.66 c	58.40 ± 0.94 c	894.76 ± 61.76 b	865.35 ± 102.23 b	53.23 ± 1.51 c	55.44 ± 3.47 c	1190.80 ± 86.05 a	1114.80 ± 73.02 a
Total ω-6	654.58 ± 5.60 b	767.95 ± 8.11 a	548.78 ± 28.01 c	467.70 ± 25.30 c	733.25 ± 21.76 ab	730.29 ± 28.96 ab	772.74 ± 49.35 a	821.99 ± 58.79 a
Saturated fatty acids (SFA)	12,533.89 ± 130.45 bc	14,750.05 ± 152.21 a	11,353.24 ± 505.99 cd	10,582.23 ± 716 d	14,107.12 ± 460.99 ab	14,366.42 ± 879.43 ab	13,752.27 ± 746.68 ab	145,029.57 ± 1010.38 a
Monounsaturated fatty acids (MUFA)	6456.17 ± 54.99 b	7551.44 ± 78.89 a	5469.07 ± 221.59 c	5051.99 ± 315.66 c	7261.04 ± 251.89 ab	7380.69 ± 407.77 ab	7140.27 ± 387.76 ab	7476.23 ± 473.488 a
Polyunsaturated fatty acids (PUFA)	703.19 ± 6.25 c	826.35 ± 8.75 c	1443.54 ± 89.76 b	1333.05 ± 125.75 b	786.48 ± 23.26 c	785.74 ± 32.41 c	1963.54 ± 133.79 a	1936.79 ± 131.78 a
Total fatty acids	19,693.26 ± 191.51 bc	23,127.85 ± 239.28 a	18,255.87 ± 816.67 c	16,967.28 ± 1155.56 c	22,154.64 ± 735.78 ab	22,532.86 ± 1319.25 ab	22,856.09 ± 1266.02 a	23,915.98 ± 1615.57 a

N.D., not detected. Moisture: Control 2.00%, Prob 2.00%, FO 0.77%, Prob + FO 0.99%, Sw 1.70%, Sw + Prob 1.70%, Sw + FO 1.82%, Sw + Prob + FO 1.13%. Treatments: Control = milk chocolate formulation, Prob = milk chocolate + probiotics, FO = milk chocolate + fish oil, Prob + FO = milk chocolate + probiotics + fish oil, Sw = isomalt + stevia, Sw + Prob = isomalt + stevia + probiotics, Sw + FO = isomalt + stevia + fish oil, Sw + Prob + FO = isomalt + stevia + probiotics + fish oil. Values with different letters within the same row indicate statically significant difference by the LDS test (*p* < 0.05). Values represent the mean of 3 replicates with their standard error. Sw, sweetener; FO, fish oil; Prob, probiotic; NS, non-significant.

**Table 4:** Sensory acceptability values of milk chocolate and sugar-free milk chocolate formulations with added probiotics and fish oil.

Sample	Appearance ^a^	Flavor ^a^	Texture ^a^	Overall Acceptability ^a^
Control	8.25 ± 0.07 a	7.07 ± 0.11 a	7.49 ± 0.09 ab	7.21 ± 0.09 a
Prob	7.22 ± 0.14 bcd	6.93 ± 0.18 a	7.63 ± 0.46 a	7.035 ± 0.15 ab
FO	6.96 ± 0.17 d	4.60 ± 0.21 d	5.409 ± 0.20 d	4.75 ± 0.21 e
Prob + FO	7.02 ± 0.16 cd	4.63 ± 0.20 d	5.48 ± 0.20 d	5.035 ± 0.21 e
Sw	7.34 ± 0.13 bc	6.39 ± 0.17 b	6.97 ± 0.14 bc	6.56 ± 0.17 c
Sw + Prob	7.49 ± 0.13 b	6.24 ± 0.17 b	6.86 ± 0.14 c	6.57 ± 0.15 bc
Sw + FO	7.15 ± 0.15 bcd	5.07 ± 0.21 cd	6.66 ± 0.18 c	5.69 ± 0.19 d
Sw + Prob + FO	7.15 ± 0.15 bcd	5.07 ± 0.20 c	6.59 ± 0.17 c	5.67 ± 0.19 d
Significance ^b^				
Sw	NS	NS	NS	NS
FO	**	***	***	***
Prob	NS	NS	NS	NS
Sw*FO	NS	***	***	***
Sw*Prob	NS	NS	NS	NS
Prob*FO	NS	NS	NS	NS
Sw*FO*Prob	NS	NS	NS	NS

Treatments: Control = milk chocolate formulation, Prob = milk chocolate + probiotics, FO = milk chocolate + fish oil, Prob + FO = milk chocolate + probiotics + fish oil, Sw = isomalt + stevia, Sw + Prob = isomalt + stevia + probiotics, Sw + FO = isomalt + stevia + fish oil, Sw + Prob + FO = isomalt + stevia + probiotics + fish oil. ^a^ Values with different letters within the same column indicate statically significant difference by the LSD test (*p* < 0.05). ^b^ Asterisks indicate significant difference from a full factorial analysis of variance showing the main effects and interactions of the variables evaluated: ** *p* < 0.01, *** *p* < 0.001. Sw, sweetener; FO, fish oil; Prob, probiotic; NS, non-significant.

## Data Availability

The data presented in this study are available on request from the corresponding author.

## Supplementary Materials

The following are available online at https://www.mdpi.com/article/10.3390/foods10081866/s1, Table S1: Fatty acid profile (mg fatty acid per 100 g sample FW^−1^) of fish oil used as a source of ω-3 polyunsaturated fatty acids.
