# Supplementary material for: Sugar-Free Milk Chocolate as a Carrier of Omega-3 Polyunsaturated Fatty Acids and Probiotics: A Potential Functional Food for the Diabetic Population
Source: Foods. 2021 Aug 12;10(8):1866. doi: 10.3390/foods10081866 (PMC8392059; doi:10.3390/foods10081866)
Supplement: Supplementary file 1 [file foods-10-01866-s001.zip › foods-1297850-supplementary.pdf]

foods

MDPI

Supplementary Materials

Sugar-Free Milk Chocolate as a Carrier of Omega-3 Polyunsaturated Fatty Acids and Probiotics: A Potential Functional Food for the Diabetic Population

Andrea R. Gómez-Fernández [1], Paulinna Faccinetto-Beltrán [1], Norma E. Orozco-Sánchez [2], Esther Pérez-Carrillo [3], Luis Martín Marín-Obispo [3], Carmen Hernández-Brenes [3], Arlette Santacruz [3] and Daniel A. Jacobo-Velázquez [1,*]

[1] Tecnologico de Monterrey, Escuela de Ingeniería y Ciencias, Av. General Ramón Corona 2514, Zapopan 45201, Mexico; andrea.rebeca.gf@gmail.com (A.R.G.-F.); paulinnafb1995@gmail.com (P.F.-B.)
[2] Escuela Mexicana de Confitería y Chocolatería, Melchor Ocampo 926, San Luis Potosi 78280, Mexico; ccinvestigacionc@gmail.com
[3] Tecnologico de Monterrey, Escuela de Ingeniería y Ciencias, Av. Eugenio Garza Sada 2501, Monterrey 64849, Mexico; perez.carrillo@tec.mx (E.P.-C.); lmmarin@live.com.mx (L.M.M.-O.); chbrenes@tec.mx (C.H.-B.); asantacruz@tec.mx (A.S.)
* Correspondence: djacobov@tec.mx; Tel.: +52-312-119-1650

Table S1. Fatty acid profile (mg fatty acid per 100 g sample FW^{-1}) of fish oil used as source of ω-3 polyunsaturated fatty acids.

**Fatty acid**	**Fish oil (mg fatty acid/100 g FW^{-1})**
Octanoic acid (C8:0)	183.6 ± 14.6
Decanoic acid (C10:0)	N.D.
Lauric acid (C12:0)	98.3 ± 1.7
Myristic acid (C14:0)	8375.8 ± 22.2
Pentadecanoic acid (C15:0)	759.4 ± 6.9
Palmitic acid (C16:0)	16,842.4 ± 62.0
Heptadecanoic acid (C17:0)	683.0 ± 6.3
Stearic acid (C18:0)	3283.4 ± 9.5
Arachidic acid (C20:0)	178.6 ± 2.7
Behenic acid (C22:0)	149.8 ± 3.0
Lignoceric acid (C24:0)	165.9 ± 1.8
Myristoleic acid (C14:1)	59.0 ± 2.2
Palmitoleic acid (C16:1)	11,325.6 ± 19.1
Oleic Acid (C18:1)	4907.2 ± 26.6
Vaccenic acid (C18:1)	2906.6 ± 12.9
Eicosenoic acid (C20:1)	498.5 ± 3.2
Nervonic acid (C24:1)	399.5 ± 1.6
Linoleic acid (C18:2)	1842.2 ± 47.9
Gamma Linolenic acid (C18:3)	268.3 ± 5.0
Alpha Linolenic acid (C18:3)	2009.1 ± 6.7
Stearidionic acid (C18:4)	3458.1 ± 18.5
Eicosadienoic acid (C20:2)	458.7 ± 1.8
Homo-gamma-linolenic acid (C20:3)	282.5 ± 1.4
Dihomogamma linolenic acid (C20:3)	372.9 ± 22.5
Arachidonic acid (C20:4)	851.2 ± 9.1
Eicosapentaenoic acid (C20:5)	12,862.1 ± 17.8
Docosapentaenoic acid n-6 (C22:5)	331.1 ± 21.1
Docosapentaenoic acid n-3 (C22:5)	1888.1 ± 25.5
Docosahexaenoic acid (C22:6)	14,122.2 ± 27.0
Total ω-3	34,712.6 ± 57.3
Total ω-6	4034.0 ± 29.6
Saturated fatty acids (SFA)	30,720.2 ± 103.6
Monounsaturated fatty acids (MUFA)	20,096.3 ± 55.2
Polyunsaturated fatty acids (PUFA)	38,746.6 ± 45.8
**Total fatty acids**	**89,563.1 ± 189.0**

N.D. = not detected.
